# Twenty-first Annual Report of the Managers of the New York State Lunatic Asylum, for the Year 1863

**Published:** 1864-04

**Authors:** 


Twenty-First Annual Report of the Managers of the State Lunatic Asylum, for the year 1863. Utica, New York.

To those interested in the statistics and treatment of mental maladies the above annual reports are of much interest and value; especially the last named, which relates to one of the oldest and largest institutions for the treatment of the insane in our country.

